# Rheumatoid Vasculitis Involving Gastrointestinal Tract Pre-dating Rheumatoid Arthritis: A Case Study and Literature Review

**DOI:** 10.7759/cureus.67346

**Published:** 2024-08-20

**Authors:** Nanditha Karra, Sumith Mustyala, Sritej Reddy Meghi, FNU Safa, Siri Sanmayi Medicherla, Ajay Goud Nattala, Jai Hind Reddy Nalla, Rajendraprasad Saligommula

**Affiliations:** 1 Internal Medicine, Osmania Medical College, Hyderabad, IND; 2 Internal Medicine, Mamata Academy of Medical Sciences, Hyderabad, IND

**Keywords:** erosive gastroduodenitis, small vessel vasculitis, autoimmune vasculitis, vasculitis, rheumatoid arthritis, rheumatoid vasculitis

## Abstract

Rheumatoid vasculitis (RV) constitutes a rare but serious complication of rheumatoid arthritis (RA), characterized by the inflammation of small and medium-sized blood vessels. We present a case of a 34-year-old male with chronic alcoholism who presented with epigastric pain, hematemesis, purpuric rashes, and multiple joint swelling and pain, without prior RA diagnosis. Abdominal angiography was unremarkable, ruling out Polyarteritis Nodosa (PAN) and Mallory Weiss tear. Upper GI endoscopy revealed erosive gastroduodenitis, and biopsies confirmed small vessel leukocytoclastic vasculitis with fibrinoid necrosis, initially raising suspicion of Henoch-Schonlein Purpura (HSP). However, positive rheumatoid factor (RF), anti-cyclic citrullinated peptide (anti-CCP) antibody, elevated inflammatory markers (erythrocyte sedimentation rate (ESR), C-reactive protein (CRP)), and negative immunoassay tests led to the diagnosis of RV. Treatment with intravenous hydrocortisone led to rapid improvement, and the patient is now being monitored on an outpatient basis with progressive recovery. This case underscores the unusual presentation of gastrointestinal involvement in RV. It highlights the importance of considering RV as a primary diagnosis, even in the absence of a prior RA diagnosis. Early recognition and intervention are critical for managing complications, emphasizing the need for a high index of suspicion in rheumatic diseases' unusual presentations.

## Introduction

Rheumatoid vasculitis (RV) is a rare but severe complication of rheumatoid arthritis (RA), affecting about 1% of patients with long-standing RA [1], predominantly males [2]. It involves systemic necrotizing vasculitis of small and medium-sized vessels and has a poor prognosis, with a nearly 40% mortality rate within five years [2], emphasizing the importance of early detection and intervention. Rheumatoid vasculitis can affect various organ systems with the cutaneous system being the most common (90% prevalence), and the peripheral nervous system being the second most common (40% prevalence) [3]. The involvement of major organs such as the heart, kidneys, bowel, and central nervous system is less common and associated with a poorer prognosis [4].

Despite a decrease in RV incidence with the use of disease-modifying anti-rheumatic drugs (DMARDs) [5], a high index of suspicion remains vital for early diagnosis and prevention of complications. The presence of skin or peripheral nervous system symptoms in rheumatoid arthritis patients should raise suspicion of RV, prompting thorough investigations. While a biopsy of the involved organ aids in the diagnosis, a complete analysis of the organ systems helps to assess the extent of involvement and the initiation of appropriate treatment. 

Here, we present a case of a male patient presenting with purpuric rashes, multiple joint pains, and hematemesis. He had no previous diagnosis of rheumatoid arthritis but is found to have rheumatoid vasculitis. The occurrence of RV involving the GI tract as a presenting feature of RA is exceedingly rare and offers valuable insights into the disease's pathophysiology.

## Case presentation

A 34-year-old male presented to the outpatient clinic with a 20-day history of developing purpuric rashes, which initially appeared on his lower limbs and gradually spread to his buttocks, back, trunk, arms, and hands and was accompanied by fever, easy fatiguability, and generalized weakness. Over the past five days, he experienced swelling and pain in various large and small joints. On the morning of his visit, he developed epigastric pain, had three episodes of hematemesis, and noticed black tarry stools. The patient also mentioned a two-year history of recurrent joint pain and swelling, which he self-medicated with Non-Steroidal Anti-Inflammatory Drugs (NSAIDs). He couldn't recall the exact number of episodes but noted that they were often accompanied by fever and malaise and typically resolved within two weeks without any rash. Additionally, he has a 15-year history of chronic alcohol consumption, drinking weekly, with his last intake occurring the night before his symptoms began. He has no significant medical or family history.

**Figure 1:**
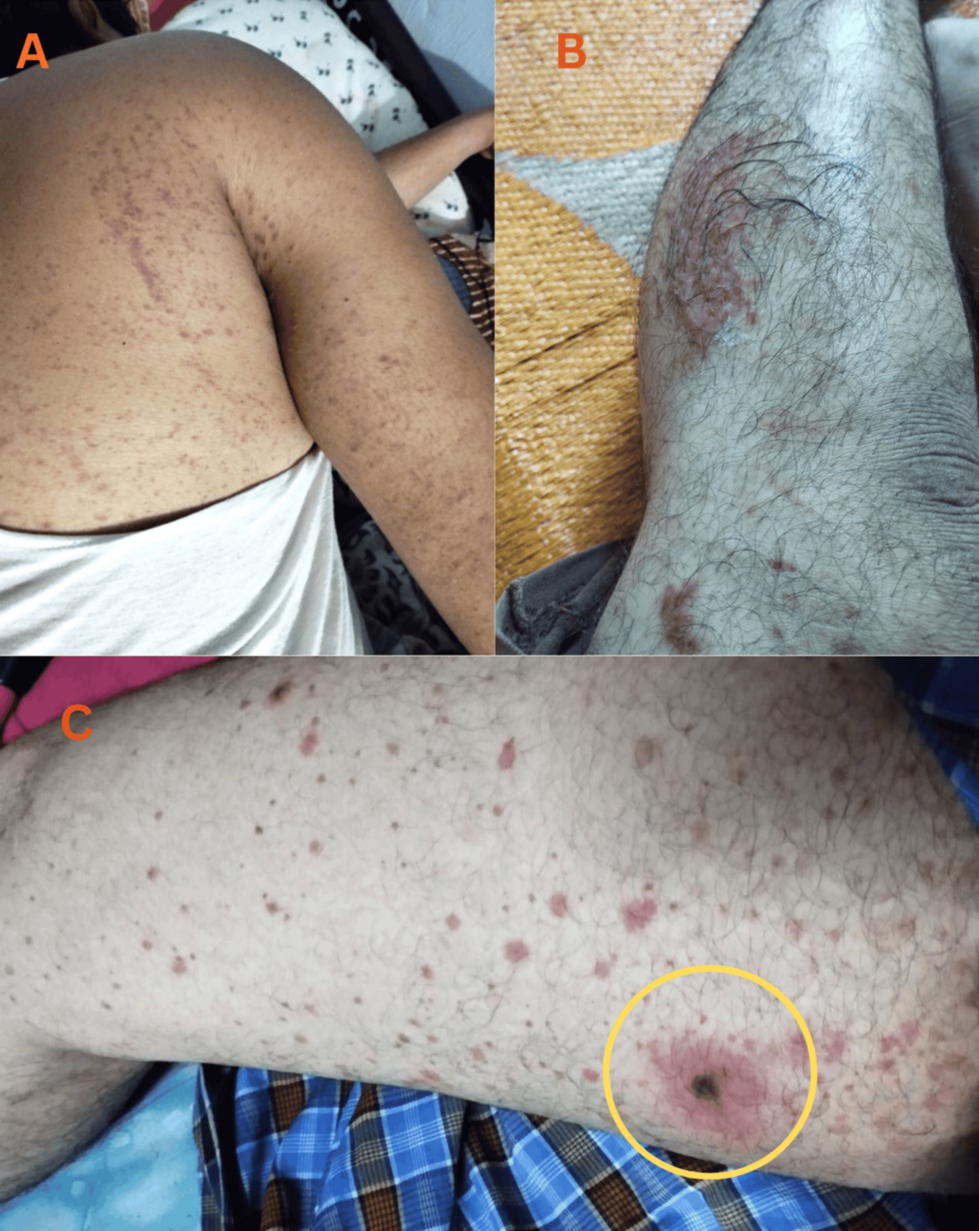
Patient images at the time of presentation A. Non-scaly, non-blanching, slightly raised, purple-colored purpura observed on the back, and the medial and posterior aspect of the arm; B. Isolated, non-scaly, non-blanching, slightly raised, purple-colored lesions observed on the lateral aspect of the calf; C. A round 2 cm ulcer (circled) with non-scaly, non-blanching, slightly raised, purple-colored purpura was observed on the medial aspect of the thigh.

**Figure 2:**
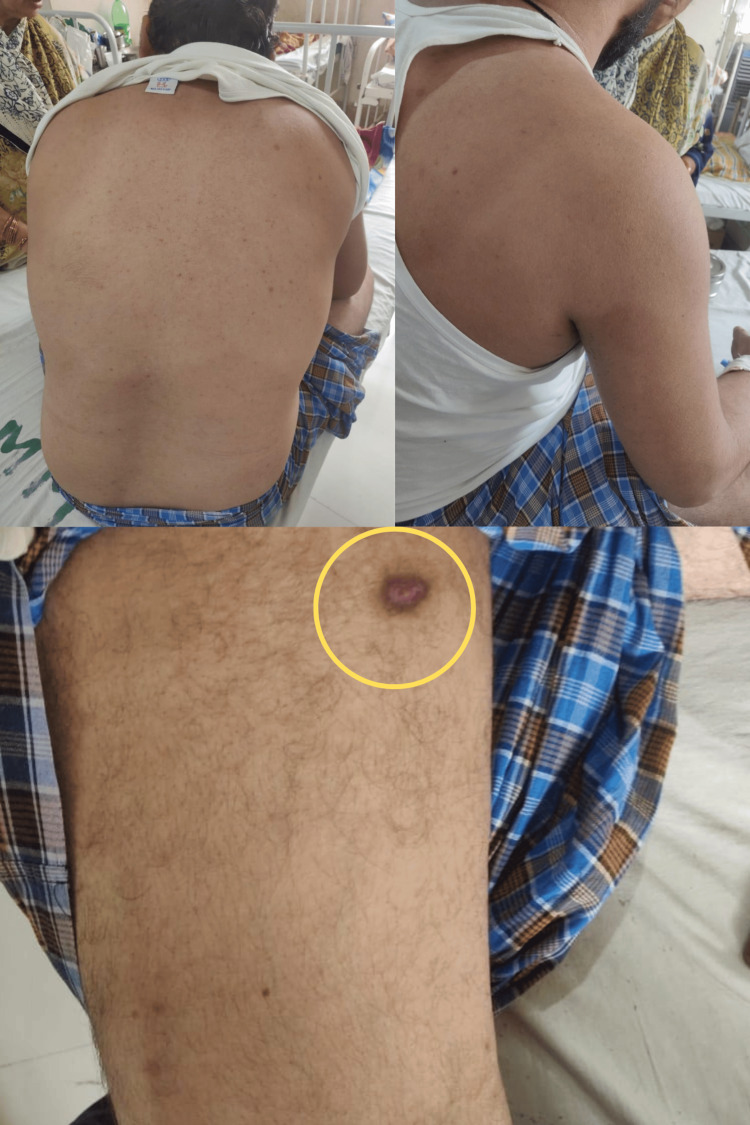
Post-treatment images The images show the disappearance of purpura in previously affected areas with a partially healed ulcer (circled) on the medial aspect of the thigh.

Upon examination, the patient appeared to be in fair condition, with all vital signs within normal ranges. He exhibited tender, non-scaly, non-blanching, palpable, purple-colored purpurae without itchiness on the buttocks, trunk, back (Figure 1A), bilateral upper (Figure 1A), and lower limbs (Figure 2B). A 2 cm round ulcer with raised, well-defined edges and a necrotic base was present on the medial aspect of the right thigh (Figure 2C), with no discharge but with induration and erythema of the surrounding skin.

There was a local rise in temperature and tenderness in the bilateral knees, shoulders, wrists, metacarpophalangeal, and proximal joints, with minimal fluctuant swelling in the knees and wrists, but no deformities or nodules in the hands or feet. The abdomen was soft, non-tender, and non-distended, with normal bowel sounds. Other systems examination was unremarkable. Investigations revealed an elevated neutrophil count, while liver, kidney, and thyroid function tests and urinalysis were normal. Tests for HIV, Hepatitis B, and C were negative. X-rays of the joints were normal, and both abdominal ultrasound and CT abdominal angiography were normal. The patient was started on symptomatic treatment, and an upper gastrointestinal (GI) endoscopy was performed, revealing erosive gastroduodenitis with a lax lower esophageal sphincter. Biopsies taken from the ulcer, purpuric skin lesions, and gastric and small intestinal mucosa indicated small vessel neutrophilic-rich leukocytoclastic vasculitis with fibrinoid necrosis. Suspecting Henoch-Schönlein Purpura (HSP), the patient was started on tranexamic acid, non-steroidal anti-inflammatory drugs (NSAIDs), and antiemetics.

Subsequent evaluations, aimed at excluding alternative diagnoses, revealed positive results for Rheumatoid Factor (RF) (Table 1) and anti-cyclic citrullinated peptide (anti-CCP) antibodies (Table 1), along with elevated levels of erythrocyte sedimentation rate (ESR), C-reactive protein (CRP), and complement components (Table 1). Conversely, the patient tested negative for antinuclear antibody (ANA), cytoplasmic antineutrophil cytoplasmic autoantibody (c-ANCA), and perinuclear antineutrophil cytoplasmic antibody (p-ANCA) (Table 1). Immunofluorescence studies for immunoglobulin (Ig)G, IgM, IgA, and C3c were negative, and the line immunoassay for ANA, conducted to rule out other autoimmune causes, was weakly positive for dsDNA antibody. 

**Table 1:** Laboratory investigations

Investigation	Results	Normal ranges
Red blood cell (RBC) count	5.53×10^6^/μL	4.3-5.9×10^6^/μL
White blood cell (WBC) count	14900/mm^3^	4500-11,000/mm^3^
Hemoglobin	14.7g/dl	13.5-17.5 g/dl
Platelets	166,000/mm^3^	150,000-400,000/mm^3^
Alanine Transaminase (ALT)	38.98 U/L	10-40 U/L
Aspartate Aminotransferase (AST)	47.77 U/L	12-38 U/L
Total Bilirubin	2.18 mg/dl	0.1-1.0 mg/dl
Direct Bilirubin	0.35 mg/dl	0.0-0.3 mg/dl
Urea	17.8 mg/dl	7-18 mg/dl
Creatinine	0.84 mg/dl	0.6-1.2 mg/dl
Na (Sodium)	132.2	136-146 mEq/L
K (Potassium)	4.45	3.5-5.0 mEq/L
Cl (Chloride)	96.0	95-105 mEq/L
Thyroid-stimulating hormone (TSH)	3.00μU/ml	0.4-4.0μU/ml
Thyroxine (T4)	1.2 ng/ml	0.9-1.7 ng/dl
Triiodothyronine (T3)	111 ng/dl	100-200 ng/dl
Prothrombin time	14 seconds	11-15 seconds
Erythrocyte Sedimentation Rate (ESR)	60mm/hr	0-15mm/hr
C-reactive Protein (CRP)	>8 mg/L	0-3 mg/L
Rheumatoid Arthritis (RA) Factor	>100 IU/ml	Less than 15-20 IU/ml
Anti-cyclic citrullinated peptide (anti-CCP) antibody	>60 EU/mL	< 20 EU/mL
Complement C3	191.9 mg/dl	90-180 mg/dl
Complement C4	91.6 mg/dl	10-40mg/dl
p-ANCA (Perinuclear anti-neutrophil cytoplasmic antibodies)	0.2IU/ml	>5.0IU/ml
c-ANCA ( cytoplasmic anti-neutrophil cytoplasmic autoantibody)	0.4IU/ml	>3.0IU/ml

The patient scored 8 on the 2010 American College of Rheumatology-European League Against Rheumatism (ACR/EULAR) classification criteria for rheumatoid arthritis [6], meeting the diagnostic criteria for the disease. Based on the EULAR score and comprehensive histopathological, clinical, and investigative findings, he was diagnosed with rheumatoid arthritis complicated by rheumatoid vasculitis. Treatment included intravenous hydrocortisone at 300 mg daily, tapered to 100 mg daily over two weeks, tranexamic acid for five days, and piperacillin-tazobactam prophylaxis for one week. By day 7, the bleeding ceased, and the patient's condition improved significantly over two weeks. The rash, joint swelling, and pain subsided except for the ulcer (Figure 2).

Upon discharge, the patient was prescribed 80 mg of prednisolone daily. At a follow-up visit one month later, he showed progressive improvement. He was referred to the rheumatology department, where all tests were repeated, and he was started on methotrexate 10 mg weekly with folic acid 5 mg weekly. He continues to be on long-term outpatient follow-up, with a plan for an endoscopy after four months of treatment.

## Discussion

Rheumatoid arthritis (RA) is a chronic systemic autoimmune disease primarily targeting joints, leading to their destruction. It can also present extra-articular manifestations, impacting various organ systems such as the skin, eyes, heart, lungs, kidneys, nervous system, gastrointestinal tract, and vascular system.

Rheumatoid vasculitis (RV) is one such rare yet catastrophic extra-articular manifestation of RA, marked by inflammation of small and medium-sized blood vessels. Although the exact pathology of RV remains unclear, it is hypothesized that the deposition of immune complexes containing rheumatoid factor (RF) and autoantibodies, such as anti-endothelial cell antibodies, in various organs causes inflammation and endothelial cell damage [5]. This damage leads to ischemia, necrosis, and organ dysfunction. Factors such as male gender, smoking, long-standing RA, specific genetic markers (HLA genotypes), and seropositivity for anti-CCP and RF increase the risk of RV [2,5,7]. Interestingly, the risk of RV generally correlates with the duration of RA, but in this case, RV was diagnosed before RA, which is uncommon.

RV affects approximately 1% of individuals with long-standing RA [1]. The skin and peripheral nervous system are the first and second most frequently involved sites respectively [3]. Cutaneous involvement can manifest as purpura, ulcers, digital ischemia, livedo reticularis, nail fold infarcts, and pyoderma gangrenosum [3,8]. Peripheral nervous system involvement may result in mononeuritis multiplex, as well as sensory, motor, or mixed neuropathies [3,8]. Gastrointestinal involvement is rare, occurring in about 10% of RV patients, with mesenteric vasculitis being the most common manifestation [4]. This can lead to serious conditions like abdominal pain, hematemesis, melena, and bowel ischemia.

There are no definitive diagnostic and classification criteria for RV. The Scott and Bacon criteria from 1984 [9], which have not been validated, suggest that RA patients should present with at least one of the following: 1. mononeuritis multiplex or peripheral neuropathy; 2. peripheral gangrene; 3. biopsy evidence of acute necrotizing arteritis plus systemic illness (e.g., fever, weight loss); or 4. active extra-articular diseases such as pleuritis, pericarditis, or scleritis, associated with biopsy findings of deep skin ulcers, digital infarcts, or vasculitis. Given the rarity of RV, there are no established management guidelines. Mild to moderate cases, limited to the skin, may be treated with moderate-dose steroids and DMARDs, whereas severe cases often require high-dose steroids combined with cyclophosphamide, followed by maintenance therapy with DMARDs [3]. Biological agents like rituximab, anti-tumor necrosis factor (TNF) drugs, tocilizumab, and abatacept have shown positive results in refractory cases [3].

In this case, a 34-year-old male with a history of chronic alcoholism presented with symptoms indicative of an upper GI bleed, purpura, and multiple joint pain and swelling. Ultrasound and CT angiogram scans were unremarkable, ruling out a Mallory-Weiss tear. An upper GI endoscopy identified erosive gastroduodenitis, explaining the bleeding and the biopsies from the purpuric lesions, ulcer, and gastric mucosa showed small vessel leukocytoclastic vasculitis with fibrinoid necrosis. The presence of purpura and histopathological findings led to the suspicion of vasculitis.

The absence of microaneurysms in the CT angiogram, normal renal function tests, and no peripheral nervous system involvement ruled out polyarteritis nodosa (PAN), leading to a provisional diagnosis of Henoch-Schönlein purpura (HSP). However, positive rheumatoid factor (RF) and anti-cyclic citrullinated peptide (anti-CCP) antibodies, along with elevated erythrocyte sedimentation rate (ESR) and C-reactive protein (CRP), prompted a reconsideration. The patient was diagnosed with rheumatoid arthritis (RA) based on the ACR/EULAR criteria [6], and the biopsy evidence of acute necrotizing arteritis plus systemic illness (fever) fulfilled the Scott and Bacon criteria [9]. Therefore, a definitive diagnosis of RA (EULAR 8) complicated by rheumatoid vasculitis (RV) was made.

This case is significant for two main reasons: Firstly, it demonstrates that rheumatoid vasculitis (RV) can precede rheumatoid arthritis (RA). To our knowledge, there are very few documented cases where RV predates or appears early in the course of RA (Table 2).

**Table 2:** Case studies of rheumatoid vasculitis predating or evolving early in rheumatoid arthritis RV: Rheumatoid vasculitis; RA: Rheumatoid arthritis

	Case Title	Authors and Year of Publication	Case description	The time between RA and RV Diagnosis
1	Vasculitis, an early unusual presentation of rheumatoid arthritis: a case report.	Kashyap et al. (2024) [5]	A 70-year-old lady presented with blackish discoloration of the tips of her fingers and toes and was diagnosed with early onset RV in RA based on laboratory investigations and clinical features.	None (RV was the presenting feature of RA)
2	Acute onset rheumatoid vasculitis with polyarthritis and erythema: a case report	Amao et al. (2023) [10]	A 70-year-old woman presented with fever, acute pain, and a mildly swollen right knee. She had general malaise, arthralgia, and severe tenderness in her shoulders, wrists, hands, and thighs. Her extremities showed palpable erythema and purpura. Positive rheumatoid factor and an ACR/EULAR 2010 score of eight led to a diagnosis of elderly-onset rheumatoid arthritis. A biopsy of erythema on her left lower leg revealed rheumatoid vasculitis.	None (RV was the presenting feature of RA)
3	Rheumatoid vasculitis, an uncommon complication of non-deforming rheumatoid arthritis: a case report.	Taha et al.(2022) [2]	An 18-year-old female presented with right fingertip ulceration, black discoloration, and bilateral wrist or metacarpophalangeal joint pain for five months. She had high anti-CCP, a doubtful rheumatoid factor titer, and an ANA titer of 1:320 with coarse nucleated cells. A definitive diagnosis of rheumatoid arthritis complicated by rheumatoid vasculitis was made.	None (RV was the presenting feature of RA)
4	Rheumatoid vasculitis as an initial presentation of rheumatoid arthritis	Lokineni et al. (2021) [11]	A 51-year-old Caucasian man presented with shortness of breath, progressive extremity pain and swelling, and an erythematous rash on his abdomen and thighs. He had had joint pain in his hands for six months, unresponsive to NSAIDs. Elevated inflammatory markers, positive RF and CCP antibodies, sensorimotor neuropathy with axonal degeneration, and skin biopsy findings confirmed a rheumatoid arthritis flare due to rheumatoid vasculitis.	None (RV was the presenting feature of RA)
5	Rheumatoid vasculitis: is it always a late manifestation of rheumatoid arthritis?	Anwar et al. (2019) [12]	A 44-year-old male had lower extremity swelling, discoloration, and ulceration, along with weakness, hand arthralgias, fatigue, weight loss, and lower extremity fluid collections. Examination revealed joint swelling, hand contractures, and muscle wasting. Serological tests confirmed rheumatoid arthritis and a skin biopsy showed features of rheumatoid vasculitis.	None (RV was the presenting feature of RA)
6	A case of rheumatoid vasculitis involving hepatic artery in early rheumatoid arthritis	Lee et al. (2017) [4]	A 72-year-old woman with two months of polyarthralgia was diagnosed with early rheumatoid arthritis. She also had livedo reticularis and atypical liver dysfunction. A liver biopsy revealed arteritis of a medium-sized hepatic artery. After ruling out other systemic causes, she was diagnosed with rheumatoid vasculitis involving hepatic arteries based on the Bacon and Scott criteria.	None (RV was the presenting feature of RA)
7	Can rheumatoid vasculitis predate a diagnosis of rheumatoid arthritis?	Sacks and Steuer (2017) [13]	A 38-year-old man initially presented with symptoms resembling polyarteritis nodosa (PAN). After six years of treatment, he developed rheumatoid arthritis (RA), suggesting that the initial diagnosis was incorrect and that he had rheumatoid vasculitis (RV) rather than PAN.	6Years
8	Pulmonary vasculitis as the first manifestation of rheumatoid arthritis	Tourin et al. (2013) [14]	A 61-year-old male presented with pulmonary vasculitis and subsequently developed symmetric polyarthritis with positive anti-CCP antibodies.	2 Months
9	Vasculitis of the gallbladder in early rheumatoid arthritis	Sandhu and Choy (2013) [15]	A 74-year-old man presented with acute cholecystitis within a week of being diagnosed with rheumatoid arthritis (RA). cholecystectomy was done and the histopathological examination of the gallbladder revealed evidence of small vessel vasculitis and rheumatoid nodules, indicating the presence of rheumatoid vasculitis (RV).	1 week
10	A case of rheumatoid vasculitis involving the gastrointestinal tract in early disease.	Parker and Chattopadhyay (2007) [15]	A 63-year-old man developed bilateral carpal tunnel syndrome, mononeuritis of both sciatic nerves, and inflammatory myositis six months after his rheumatoid arthritis diagnosis. Systemic rheumatoid vasculitis (RV) was diagnosed based on clinical features and lab tests. Two months later, he had a small bowel perforation, with histology showing endarteritis obliterans of medium-sized arteries and recanalization, indicative of end-stage RV.	6 Months

Secondly, it underscores the rare occurrence of RV affecting the gastrointestinal (GI) tract. There are very few documented cases where RV affected the hepatic artery [4], gallbladder [15], and small bowel [16], early in the disease process of RA. In the majority of the cases where the GI tract was involved, the patients already had well-established RA diagnoses, leading to GI infarction [17], multiple intestinal ulcers [18], and intra-abdominal hemorrhage [19] (this case also has amyloid A amyloidosis in addition to long-standing RA). Our case highlights the importance of considering RV as a potential diagnosis in early or undiagnosed RA cases, despite its rarity, as this can significantly improve patient outcomes. Additionally, it emphasizes the need to consider GI tract involvement in RV early in the disease course rather than as a diagnosis of exclusion to enable early management and prevent complications.

## Conclusions

This case highlights the rare occurrence of rheumatoid vasculitis involving the gastrointestinal tract before the onset of rheumatoid arthritis. It underscores the diagnostic challenge of identifying RV, particularly when it precedes the typical RA symptoms. The case also emphasizes the necessity for clinicians to maintain a high index of suspicion for RV in patients presenting with vasculitis and also to consider gastrointestinal involvement early in the disease course. This approach is crucial for timely diagnosis and appropriate therapeutic intervention. Moreover, the case illustrates the importance of early diagnosis and treatment in improving patient outcomes and reducing mortality, especially in atypical presentations of RV. Further research and case studies are needed to enhance the understanding and formulate management strategies for RVs with gastrointestinal involvement.
